# A Hybrid Model of the Role of VEGF Binding in Endothelial Cell Migration and Capillary Formation

**DOI:** 10.3389/fonc.2013.00102

**Published:** 2013-05-10

**Authors:** Harsh V. Jain, Trachette L. Jackson

**Affiliations:** ^1^Department of Mathematics, Florida State UniversityTallahassee, FL, USA; ^2^Department of Mathematics, University of MichiganAnn Arbor, MI, USA

**Keywords:** mathematical model, angiogenesis, VEGF binding dynamics, endothelial cell migration, hybrid modeling

## Abstract

Vascular endothelial growth factor (VEGF) is the most studied family of soluble, secreted mediators of endothelial cell migration, survival, and proliferation. VEGF exerts its function by binding to specific tyrosine kinase receptors on the cell surface and transducing the effect through downstream signaling. In order to study the influence of VEGF binding on endothelial cell motion, we develop a hybrid model of VEGF-induced angiogenesis, based on the theory of reinforced random walks. The model includes the chemotactic response of endothelial cells to angiogenic factors bound to cell-surface receptors, rather than approximating this as a function of extracellular chemical concentrations. This allows us to capture biologically observed phenomena such as activation and polarization of endothelial cells in response to VEGF gradients across their lengths, as opposed to extracellular gradients throughout the tissue. We also propose a novel and more biologically reasonable functional form for the chemotactic sensitivity of endothelial cells, which is also governed by activated cell-surface receptors. This model is able to predict the threshold level of VEGF required to activate a cell to move in a directed fashion as well as an optimal VEGF concentration for motion. Model validation is achieved by comparison of simulation results directly with experimental data.

## 1 Introduction

Motility – random, directed, and collective – is a fundamental property of cells. Coordinated cellular motion leads to all physiological tissue patterns, a consequence of integration across multiple temporal and spatial scales. However, when this integration is aberrant, the properties that emerge lead to a critical bifurcation point in cancer progression: angiogenesis. Angiogenesis, the formation of new blood vessels from pre-existing ones, provides the necessary blood supply for the growth and nourishment of solid tumors beyond a few millimeters in diameter (Hanahan and Weinberg, 2000; Augustin et al., 2009). Tumor angiogenesis is associated with an extremely complex, yet well-ordered series of events at the center of which is the enhanced replicative potential and motility of endothelial cells (ECs) that line the inner surface of blood vessels (Folkman, 1985; Hanahan and Weinberg, 2000).

The multistep process associated with successful angiogenesis can be summarized as EC degradation of the adjacent basement membrane, migration (sprouting), proliferation, alignment, tube formation, branching that increases near the tumor leading to a brush-border, anastomosis (fusion of vessels), synthesis of new basement membrane, recruitment of parenchymal cells, network remodeling, and a return to quiescence (Folkman, 1985; Yancopoulos et al., 2000; Conway et al., 2001; Augustin et al., 2009). Precise coordination and integration of molecular, cellular, and tissue level interactions is required for angiogenesis to be successful from initiation to stabilization of a functional vascular plexus.

Under conditions of hypoxia, tumor cells induce angiogenesis by releasing a wide variety of polypeptide angiogenic factors that stimulate EC activation, survival, proliferation, migration, and maturation. Members of the vascular endothelial growth factor (VEGF) family have been identified as the predominant amongst these angiogenic factors that regulate EC phenotype (Yancopoulos et al., 2000; Jain, 2002; Ferrara, 2004; Hicklin and Ellis, 2005). VEGF has been implicated across a range of human cancer and preclinical studies have shown that VEGF stimulates survival of existing vessels, promotes new vessel growth, and contributes to vascular abnormalities such as tortuousness and hyperpermeability. The angiogenic effects of the VEGF pathway are primarily initiated through the interaction of VEGFA and its natural, endothelial cell specific receptor, VEGFR2, which is up-regulated during angiogenesis (Neufeld et al., 1999; McMahon, 2000; Conway et al., 2001). Dimerization and activation of VEGFR2 results in mitogenic, chemotactic, and prosurvival signals (Nor et al., 1999; Ferrara et al., 2003; Ferrara, 2004), which help to determine endothelial cell phenotype.

The various steps of the angiogenic cascade require endothelial cells to take on spatio-temporally varying phenotypes; that is, at any given time and at any specific spatial location within a developing sprout, ECs can have a proliferative, migratory, or quiescent phenotype. For example, tip cells are highly migratory and lead the extending sprout through the extracellular matrix (ECM), whereas stalk cells, which form the vessel lumen and recruit support cells, can be either proliferative or quiescent. It has been shown that endothelial cells compete for the tip cell position through relative levels of VEGF-receptors (Jakobsson et al., 2010). While much is known about the sequential morphogenetic processes required for angiogenesis and the growth factors that drive it, far less is known about how cellular and molecular mechanisms are coordinated to control cell motility decisions and phenotype choices. In order to advance the understanding and manipulating of the processes that occur during angiogenesis, it is critical to understand how individual cells interpret the biochemical signals that come from their unique microenvironment.

For decades, mathematical models have been employed to help address some of the pressing questions associated with tumor angiogenesis. As discussed in detail in Jackson and Zheng (2010), Zheng et al. (2013), existing models of tumor-induced angiogenesis can be characterized as continuous approaches (Balding and McElwain, 1985; Byrne and Chaplain, 1995, 1996; Anderson and Chaplain, 1998a,b; Holmes and Sleeman, 2000; Levine et al., 2001; Arakelyan et al., 2002; Sleeman and Wallis, 2002; Manoussaki, 2003; Plank and Sleeman, 2003, 2004; Plank et al., 2004; Levine and Nilsen-Hamilton, 2006; Schugart et al., 2008; Billy et al., 2009; Xue et al., 2009; Travasso et al., 2011), wherein cells are assumed to have a continuous distribution; discrete or hybrid models (Stokes and Lauffenburger, 1991; Anderson and Chaplain, 1998b; Tong and Yuan, 2001; Plank and Sleeman, 2003, 2004; Sun et al., 2005; Bartha and Rieger, 2006; Gevertz and Torquato, 2006; Frieboes et al., 2007; Milde et al., 2008; Capasso and Morale, 2009; Owen et al., 2009; Perfahl et al., 2011), wherein cells are modeled as individual agents and diffusible chemicals are modeled as a continuum; and cell-based formulations (Peirce et al., 2004; Bauer et al., 2007; Bentley et al., 2009; Qutub and Popel, 2009; Wcislo et al., 2009; Jackson and Zheng, 2010; Liu et al., 2011) wherein explicit incorporation of different properties of individual cells allows collective behavior of cell clusters to be predicted from the behavior and interactions of individual cells. Reviews of these models that appeared in or before 2009 can be found in Mantzaris et al. (2004), Peirce (2008), Qutub et al. (2009). However, these models suffer from the following limitations. Continuum descriptions of biological motion such as chemotaxis are derived by averaging quantities such as cellular and vascular densities, and therefore apply to the macroscopic behavior of a large number of cells. However, the initial stages of new capillary development requires only a small number of cells in a highly discrete arrangement, which is better described by treating cells as individual agents. Further, even when a hybrid or cell-based approach has been adopted, endothelial cell movement, and/or microvessel formation speed and direction is typically assumed to depend on extracellular chemokine concentrations, whereas it is known that cells integrate the chemical signal via receptors on their surfaces in order to make behavioral decisions (Nor et al., 1999; Ferrara et al., 2003; Ferrara, 2004).

In order to study the influence of VEGF binding on EC motion, we develop here a hybrid model of VEGF-induced angiogenesis that is based on the theory of reinforced random walks. We will include in our model, the chemotactic response of endothelial cells to angiogenic factors bound to cell-surface receptors, rather than approximating this as a function of extracellular chemical concentrations. This will allow us to capture biologically observed phenomena such as the activation and polarization of endothelial cells in response to VEGF gradients across their lengths. We will also propose a novel and more biologically reasonable functional form for the chemotactic sensitivity of cells, which is also governed by activated cell-surface receptors.

In the sections that follow, we will first develop a model to describe the motion of a single EC that has a tip cell phenotype. The motion of an EC across a 1 mm^2^ domain will be simulated, and average time taken by the cell to reach the tumor (VEGF source) computed as a function of source strength. We will then extend this model to study the early stages of blood vessel formation and sprout extension. In particular, we will capture the following growth pattern of developing sprouts, typically observed in experiments. Sprouts arising from parent vessels are observed to grow parallel to each other initially (Paweletz and Knierim, 1989), with anastomoses between tip cells and stalk cells, or between two tip cells observed to occur a certain distance into the stroma. As the developing vessels near the source of chemoattractants, new sprouts emerge in a process called sprout branching, which increases with proximity to the source. This has been described as a “brush-border” effect (Muthukkaruppan et al., 1982; Sholley et al., 1984). We will also investigate the effect on vascular development of the source strength of VEGF. Model validation will follow from a qualitative comparison of simulations with experimental data on neovascularization of the rat cornea taken from Sholley et al. (1984).

## 2 Materials and Methods

### 2.1 Model development: Single cell motion under the influence of VEGF

A vital characteristic of all cells is their ability to sense their environment and respond to it, such as motion toward or away from an external, chemical stimulus. The response of endothelial cells to a chemokine like VEGF involves the following two major steps that a mathematical description of this process needs to account for: (i) detection of the signal (via gradient of bound VEGF to cell-surface receptors) and (ii) transduction of the external signal into an internal signal that controls the pattern of movement (Mantzaris et al., 2004). The theory of reinforced random walks, where a master equation governing cell movement is derived directly from the governing biology, as opposed to discretizing a continuous equation of macroscopic motion, provides a natural framework for modeling the movement of individual endothelial cells that initiate vascular sprout development. We remark that for simplicity, the effects of the extracellular matrix on EC motion are not explicitly considered at this time so that the cell is assumed to migrate on a homogeneous and isotropic medium. Further, on the time-scale of interest, cell proliferation, and death are assumed to be negligible.

**Figure 1:**
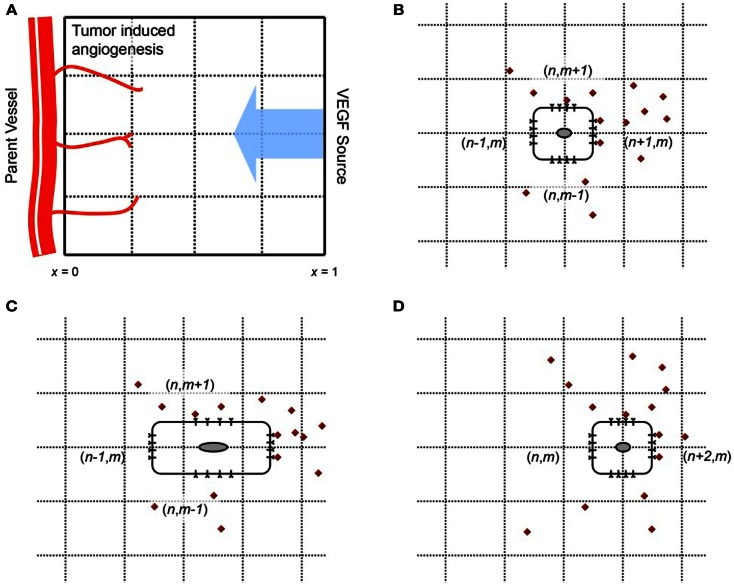
**(A)** Geometry of the model domain. A source of VEGF (e.g., a tumor) is situated at *x* = 1. VEGF diffuses toward a parent vessel located at *x* = 0, and is taken up by endothelial cells lining it. The activated cells migrate up gradients of VEGF, elongating behind them capillaries. **(B–D)** Motion of a cell on a 2-d lattice in response to VEGF stimulus. The cell begins at position (*n*, *m*) in **(B)**. VEGF molecules are shown in red. The number of activated VEGF-receptors is greatest on the cell surface at lattice site (*n* + 1*/*2, *m*), and the probability of motion in this direction is the greatest. Consequently, the cell is likely to move from site (*n*, *m*) to (*n* + 2, *m*), as shown in **(C,D)**.

We begin our model development by first simulating a single EC moving under the influence of VEGF. The EC is interpreted as a (biased) random walker that adapts its motility decisions under the influence of activated VEGF-receptors on its surface. We consider a 2-dimensional spatial domain, with a tumor located at *x* = 1 serving as the source of VEGF, and a parent vessel located at *x* = 0 providing individual ECs to begin sprout development, as shown in Figure 1A. The tumor secretes VEGF under the condition of hypoxia, which diffuses toward the parent vessel. VEGF is taken up by cells lining the parent vessel, transforming them into sprout tip cells. These migrate up its chemical gradient, pulling behind them the developing capillaries. The principal dynamics that we wish to capture with the model are the binding and uptake of VEGF by the sprout tip cells, the subsequent activation of cell-surface receptors, and the chemotactic response of the cells to this stimulation. To our knowledge, this level of molecular detail has not been implemented previously in a model of tumor-induced angiogenesis.

The cell is located initially at spatial position *x* = 0, *y* = 0.5, and will move in response to a local, cellular gradient of VEGF, which has its source at *x* = 1. A schematic of this process is shown in Figures 1B–D. Following Plank et al. (Plank and Sleeman, 2003; Plank et al., 2003, 2004), we base our spatial discretization on purely biological considerations. As per the approach developed in Plank and Sleeman (2003), Plank et al. (2003), Plank et al. (2004), Othmer and Stevens (1997), the following master equation is used to describe a biased random walk (in two dimensions) of the endothelial cell, moving under the influence of VEGF in its local environment:

(1) $$\begin{array}{ll} \frac{\partial p_{n,m}}{\partial t}= & {\hat{T}}_{n-1,m}^{H+}\left(W\right)p_{n-1,m}+{\hat{T}}_{n+1,m}^{H-}\left(W\right)p_{n+1,m}\\ & \;+{\hat{T}}_{n,m-1}^{V+}\left(W\right)p_{n,m-1}+{\hat{T}}_{n,m+1}^{V-}\left(W\right)p_{n,m+1}\\ & \;-\left({\hat{T}}_{n,m}^{H+}\left(W\right)+{\hat{T}}_{n,m}^{H-}\left(W\right)+{\hat{T}}_{n,m}^{V+}\left(W\right)\right.\\ & \;\left.+{\hat{T}}_{n,m}^{V-}\left(W\right)\right)p_{n,m}. \end{array}$$

Here, *p_n,m_*(*t*) describes the probability that a cell is at site (*n*, *m*), at time *t*. ${\hat{T}}_{n,m}^{H\pm }\left(\cdot \right),$ and ${\hat{T}}_{n,m}^{V\pm }\left(\cdot \right)$ are the transition probabilities per unit time for a one step horizontal jump to (*n* ± 1, *m*), or a one step vertical jump to (*n*, *m* ± 1) respectively. The vector *W* gives the concentration of the chemoattractant *C*, at the lattice sites. In order for the master equation to translate to the standard diffusion-chemotaxis equation for cell movement in the continuum limit, it is assumed that the dependence of transition rates at lattice site (*n*, *m*) is localized to chemoattractant concentration at sites (*n* ± 1*/*2, *m*) and (*n*, *m* ± 1*/*2). This is reasonable, since we may think of a cell present at lattice site (*n*, *m*), with its boundaries extending to half the lattice length. The cell can therefore sense the chemical concentrations at these half-lattice sites, and make a decision where to move, as illustrated in Figures 1B–D. Under these assumptions, *W* = (…, *C*_−*n*−1/2,*m*_, *C*_−*n*,*m*_, *C*_−*n*+1/2,*m*_, *C*_−*n*+1,*m*_, …).

The mean waiting time at the (*n*, *m*)th site is given by $1∕\left({\hat{T}}_{n,m}^{H+}\left(W\right)+{\hat{T}}_{n,m}^{H-}\left(W\right)+{\hat{T}}_{n,m}^{V+}\left(W\right)+{\hat{T}}_{n,m}^{V-}\left(W\right)\right).$ We make the assumption that the decision of where to move in space is independent of the decision when to move in time. Mathematically, this is equivalent to setting

(2) $${\hat{T}}_{n,m}^{H+}\left(W\right)+{\hat{T}}_{n,m}^{H-}\left(W\right)+{\hat{T}}_{n,m}^{V+}\left(W\right)+{\hat{T}}_{n,m}^{V-}\left(W\right)=k.$$

That is to say, the cell makes a decision to move (or to stay still) after a constant amount of time, *k*. One way to satisfy these assumptions is the following choice of transition probabilities, as made by Othmer and Stevens (1997)):

(3) $$\begin{array}{c} T_{n,m}^{H\pm }=\frac{1}{k}\frac{\tau \left(C_{n\pm 1∕2,m}\right)}{\begin{array}{c} \tau \left(C_{n+1∕2,m}\right)+\tau \left(C_{n-1∕2,m}\right)\\ \;+\tau \left(C_{n,m+1∕2}\right)+\tau \left(C_{n,m-1∕2}\right) \end{array}},\\ T_{n,m}^{V\pm }=\frac{1}{k}\frac{\tau \left(C_{n,m\pm 1∕2}\right)}{\begin{array}{c} \tau \left(C_{n+1∕2,m}\right)+\tau \left(C_{n-1∕2,m}\right)\\ \;+\tau \left(C_{n,m+1∕2}\right)+\tau \left(C_{n,m-1∕2}\right) \end{array}}, \end{array}$$

for some function *τ*(*C*) of the chemoattractant. The choice of the functional form for *τ*(*C*) is based on the particular form of the chemotactic sensitivity desired, and is explained in the following section. A grid of mesh size *h* is chosen, thereby fixing *x* = *nh*. Passing to the continuum limit *h* → 0 and 1/4*k* → ∞ such that *h*^2^/4*k* = *D_p_*, where *D_p_* is the diffusion coefficient of ECs, Othmer and Stevens (1997) show that the master equation (1) translates to the familiar diffusion-chemotaxis equation (4), for cell motion.

(4) $$\frac{\partial p}{\partial t}=D_{p}\Delta p-\nabla \cdot \left(p\chi \left(C\right)\nabla C\right),$$

where the chemotactic sensitivity χ(*C*) = *D_p_*(ln *τ*(*C*))’. To get a completely discretized model of the motion of the cell, the time derivative of *p* in equation (1) is approximated by a simple forward difference scheme, with *k* as the time step, given by

(5) $$k=\frac{h^{2}}{4D_{p}}.$$

A diagrammatic representation of the motion of the cell is shown in Figure 1. The cell starts out at time *t* at the lattice site (*n*, *m*) (Figure 1B). Endothelial cells are large enough to detect gradients of chemoattractants across their length, which is typically 20 μm (Vadapalli et al., 2000). In contrast to existing models of cellular chemotaxis, in which cells typically respond to free, extracellular chemokine concentrations, or their gradients in the surrounding tissue, the model developed here will capture the response of ECs to VEGF that is bound to cell-surface receptors. VEGF-VEGFR2 binding is known to be the signal that initiates endothelial motility, therefore incorporating this molecular response is important for a realistic description of cell motion.

The cell detects *bio-available* VEGF by taking it up at the half-lattice sites (Figure 1B). The model will thus make the crucial distinction between VEGF that is free to bind to the cell, versus VEGF that might be sequestered in the underlying extracellular matrix, unavailable to the cell. Based on the numbers of activated receptors at its four sides, the cell becomes polarized and attains a bias in a particular direction. It correspondingly elongates in this direction (Figure 1C). Finally, the rear of the cell detaches from the underlying matrix and contracts, and the cell has now moved to the site (*n* + 1, *m*) (Figure 1D). We remark that it has been observed experimentally that ECs may respond to chemoattractant concentration differences of as small as 2% across their length, frequently at concentrations at which molecular fluctuations are significant (Mantzaris et al., 2004). In our model, fluctuations of the order of 100 molecules of VEGF per cell are significant enough to alter its polarization, and hence its direction of motion.

### 2.2 A novel chemotactic sensitivity function

An important difference that sets this model apart from those preceding it, is the choice of the chemotactic sensitivity function χ(*C*). Various choices have been proposed thus far in the modeling literature for χ(*C*), for a review of the most commonly used functional forms (see Ford and Lauffenburger, 1991). The simplest choice is to assume that the chemotactic sensitivity is constant, χ(*C*) = χ_0_ (Keller and Segel, 1971a,b). However, this implies that the chemotactic sensitivity is unchanging in the presence of the chemoattractant, and does not account for the desensitization of cells which has been experimentally observed to occur in regions of high chemokine concentrations (Kuppuswamy and Pike, 1989; Wang et al., 2000; Kurt et al., 2001). To overcome this, Lapidus and Schiller (1976), and later Murray (2003) used the functional form χ(*C*) = χ_0_/(*K* + *C*)^2^, also known as the receptor-kinetic law. This has the advantage that it is able to account for the desensitization of receptors when *c* is large. Yet another popular phenomenological choice is χ(*C*) = χ_0_/(*K* + *C*), where *K* is the dissociation constant of the chemokine binding to the receptors (Balding and McElwain, 1985; Anderson and Chaplain, 1998b; Plank et al., 2003).

Although the choices mentioned above have been widely used in the angiogenesis literature, there are a few biological issues that these choices do not address. Firstly, they indicate that when no chemokine is present at a site, the chemotactic sensitivity is the greatest, and that the sensitivity decreases as chemokine concentrations increase. However, there is experimental evidence which points toward the existence of a minimum threshold level of chemical stimulus required for the cell-surface receptors to become activated, and for the cell to start moving in a directed fashion (Favier et al., 2006; Liu et al., 2006). This threshold has been incorporated in the cell-based model of tumor angiogenesis by Bauer et al. (2007). Secondly, the above functions do not account for the fact that the amount of chemokine required to desensitize cells depends on the concentration of cells present at the lattice site. For instance, while 10 fg of VEGF is enough to desensitize a single EC, it is not enough for 10 cells. Finally, for an external chemical signal to elicit a chemotactic response from a cell, it needs to be detected by the cell, and transduced into an internal signal controlling cell motion. Neutrophils have been shown to sense chemical gradients of 1% across their lengths, under optimal conditions (Wang et al., 2004; Levine et al., 2006), while this number can be as low as 0.1% for axons (Wang et al., 2004). In general, eukaryotic cells are reported to be able to polarize and migrate in a directed fashion in alignment with chemical gradients of about 2% across their lengths (Franz et al., 2002). It is therefore biologically more reasonable to assume that the chemotactic response of cells is dependent on the gradients of activated receptor complexes formed on the cell surface when the chemokine binds to its receptors, rather than gradients of free chemokine concentration throughout the tissue.

To address these concerns, we propose that the chemotactic sensitivity function should in fact be a function of the activated receptor concentration, *A*. In this case, equation (4) for the motion of a cell in 2 dimensions transforms to the following:

(6) $$\frac{\partial p}{\partial t}=D_{p}\Delta p-\nabla \cdot \left(\chi \left(A\right)p\nabla A\right).$$

Correspondingly, the transition probabilities in equation (1) will now be functions of concentration of activated receptor complexes on the cell surface *a* and not extracellular VEGF. That is,

(7) $$\begin{array}{c} T_{n,m}^{H\pm }=\frac{1}{k}\frac{\tau \left(A_{n\pm 1∕2,m}\right)}{\begin{array}{c} \tau \left(A_{n+1∕2,m}\right)+\tau \left(A_{n-1∕2,m}\right)\\ \;+\tau \left(A_{n,m+1∕2}\right)+\tau \left(A_{n,m-1∕2}\right) \end{array}},\\ T_{n,m}^{V\pm }=\frac{1}{k}\frac{\tau \left(A_{n,m\pm 1∕2}\right)}{\begin{array}{c} \tau \left(A_{n+1∕2,m}\right)+\tau \left(A_{n-1∕2,m}\right)\\ \;+\tau \left(A_{n,m+1∕2}\right)+\tau \left(A_{n,m-1∕2}\right) \end{array}}. \end{array}$$

We have to add equations for the binding of chemokine to their cell-surface receptors, which will need to be solved wherever a cell is present (see Section 2.3). Biologically, the chemotactic sensitivity χ(*A*)▽*A* can be interpreted by breaking it down as follows: a velocity χ(*A*) imparted to the cell due to the presence of bound chemokine on its surface, and a gradient ▽*A* which governs the direction of motion. This gradient simply means that the cell is able to sense the amount of chemokine bound to its various faces, and is correspondingly able to align itself for motion in this direction. Therefore, *a* is in fact taken to be the amount of activated receptors *per* *cell face*. We choose a velocity function that satisfies the requirements that there can be no chemotaxis in the absence of a signal, and that the cell gets desensitized in the presence of excess signal. One such functional form is:

(8) $$\chi \left(A\right)=\chi_{0}Ae^{-A∕K}.$$

The maximum of this function occurs at *A* = *K*, while its maximum value is given by χ_0_*Ke*^−1^. In order for this choice to be consistent with the discrete formulation, the function *τ*(*A*), from equation (3) must be taken as follows;

(9) $$\tau \left(A\right)=\exp\left[\frac{\chi_{0}K}{D_{p}}\left(K-\left(K+A\right)e^{-A∕K}\right)\right].$$

**Figure 2:**
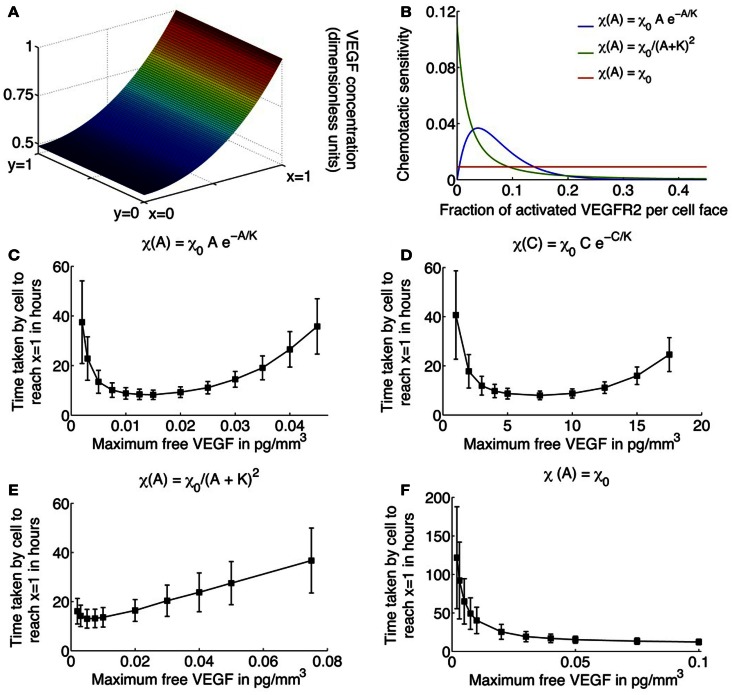
**(A)** Typical profile of unbound VEGF, the source of which is located at *x* = 1. **(B)** Various choices for the chemotactic sensitivity of an endothelial cell to VEGF bound to its surface receptors, as a function of the fraction of activated VEGFR2 per cell face. **(C–F)** Average migration times (in hours) for a single cell to travel across a 1 mm × 1 mm domain as a function of increasing the maximum free VEGF concentration at a lattice site, for various choices of the chemotactic sensitivity function. Cells are assumed to respond to activated VEGFR2 on their surfaces, with chemotactic sensitivity taken as: **(C)** as proposed in equation (8); **(D)** cells are assumed to respond to free VEGF with chemotactic sensitivity as defined in equation (8) with activated receptor concentration *A* replaced by free VEGF concentration *C*; **(E)** receptor-kinetic law, for which *τ*(*A*) = *e*^χ0*A/*(*KDp*(*K*+*A*))^, χ_0_ = 0.4416 (pg/mm^3^) mm^2^/h, *K* = 2 pg/mm^3^; and **(F)** constant, χ_0_ = 0.0046 mm^2^/(pg/mm^3^)/h.

The parameters χ_0_ and *K* are unknown in our model formulation, and would ideally be determined from experimental observations. *K* specifies the fractional occupancy of the receptors on the cell surface at which its chemotactic response is the greatest, while χ_0_ determines the maximum value of this response. Here, values of these parameters are chosen to produce biologically realistic simulation results. Figure 2B plots the chemotactic sensitivity equations (8) as a function of the fraction of activated receptors on a cell face, for a particular choice of *K* and χ_0_. We can see that at zero fractional activation, the cell remains inactive. The sensitivity peaks at 5% fractional activation of receptors, and decays thereafter. Also shown for comparison are the receptor-kinetic law, and constant chemotactic sensitivity.

### 2.3 VEGF-VEGFR2 binding dynamics

We now describe the equations governing the rates of change of the concentrations of free VEGF (*C*), free VEGFR2 (*R*), VEGF-VERFR2 monomers (*M*), and activated VEGF-VEGFR2 dimer complexes (*A*). Beginning with free VEGF, we assume that the processes of diffusion and natural decay dominate the dynamics, which are represented by the reaction-diffusion equation:

(10) $$\frac{\partial C}{\partial t}=D_{c}\Delta C-\alpha_{c}C-f\left(p\right)C.$$

Here, *D_c_* is the diffusion coefficient of VEGF, and α*_c_* is its rate of decay in tissue. The uptake of VEGF by the migrating EC has also been accounted for via the term *f*(*p*)*C*, which is derived in the following discussion. As in Anderson and Chaplain (1998b), a line source of tumor cells is assumed at *x* = 1 that produces VEGF at a constant rate, so that *C*(1, *y*, *t*) = *C*_0_. At each of the remaining domain boundaries *x* = 0 and *y* = 0, 1, a no-flux condition is imposed on free VEGF. Figure 2A shows the distribution of VEGF, expressed in non-dimensional terms, as determined by equation (10) across the domain.

At each half-lattice site surrounding a site occupied by an EC, free extracellular VEGF (*C*) binds to free cell-surface receptors, VEGFR2 (*R*), to form activated dimerized receptor complexes (*A*). Following Jain et al. (2008), we assume ligand-induced dimerization to be the dominant mechanism by which VEGF activates VEGFR2, as represented by the following chemical reactions:

$$\begin{array}{ccc} C & + & R\overset{k_{f1}}{\underset{k_{r1}}{⇌}}M\\ M & + & R\overset{k_{f2}}{\underset{k_{r2}}{⇌}}A\\ & & A\overset{k_{p}}{\to }2R \end{array}$$

the rates of forward reactions are indicated above the reaction arrows, while those of reverse reactions are indicated below the reaction arrow.

This reaction diagram can be converted to the following system of differential equations using principles of mass balance:

(11) $$\begin{array}{lll} \frac{dC}{dt} & =-2\eta_{1}k_{f1}CR+\eta_{2}k_{r1}M, & \end{array}$$

(12) $$\begin{array}{lll} \frac{dR}{dt} & =-2k_{f1}CR+\eta_{3}k_{r1}M-k_{f2}MR+2\eta_{4}k_{r2}A+2\eta_{4}k_{p}A, & \end{array}$$

(13) $$\begin{array}{lll} \frac{dM}{dt} & =2\eta_{5}k_{f1}CR+k_{r1}M-\eta_{5}k_{f2}MR+2\eta_{6}k_{r2}A, & \end{array}$$

(14) $$\begin{array}{lll} \frac{dA}{dt} & =\eta_{7}k_{f2}MR-2k_{r2}A-k_{p}A. & \end{array}$$

The multiplicative factor 2 in some of the equations accounts for the possibility that there may be two ways for that product to form. The constants η*_i_* represent the ratios of weights of different molecules and have been introduced to express chemical concentrations in units of pg/mm^3^. The values of these constants are given in Table 1 and have been estimated from Ferrara et al. (2003), Stewart et al. (2003).

**Table 1:** **Parameter values relating to the molecular weights of VEGF and VEGFR2**.

Parameter	Value	Units
η_0_	0.1101	pg VEGF per pg VEGFR2-VEGF-VEGFR2
η_1_	0.2250	pg VEGF per pg VEGFR2
η_2_	0.1837	pg VEGF per pg VEGFR2-VEGF
η_3_	0.8163	pg VEGFR2 per pg VEGFR2-VEGF
η_4_	0.4494	pg VEGFR2 per pg VEGFR2-VEGF-VEGFR2
η_5_	1.2250	pg VEGFR2-VEGF per pg VEGFR2
η_6_	0.5506	pg VEGFR2-VEGF per pg VEGFR2-VEGF-VEGFR2
η_7_	2.2250	pg VEGFR2-VEGF-VEGFR2 per pg VEGFR2

Since EC migration and sprout elongation occurs on a time-scale of several hours to days, and the biochemical reactions equations (11–14) occur on a time-scale of several scones to minutes, we assume that the VEGF-receptor complex concentrations *M* and *A* are at quasi steady state. This is equivalent to setting the left hand sides of equations (13) and (14) to zero, and solving for *M* and *A*. Further, by conservation of total receptor number, we have *R* + η_3_*M* + 2η_4_*A* = *R_f_N*, where *R_f_* is the total number of receptors per EC face and *N* is the number of ECs (*N* = 1 in the case of single cell migration, and *N* = the total number of tip cells in the case of capillary formation). We therefore deduce that at quasi steady state,

(15) $$A=\frac{\begin{array}{c} -2\alpha \delta -\gamma +\theta \beta \\ \;+\sqrt{{\left(2\alpha \delta +\gamma -\theta \beta \right)}^{2}+4\alpha \beta \left(\eta_{4}+\delta \right)\left(\theta -\alpha \right)} \end{array}}{2\beta \left(\eta_{4}+\delta \right)},$$

where,

(16) $$\begin{array}{llll} \alpha & =\frac{k_{r1}R_{f}}{2k_{f1}C+k_{r1}},\;\beta =\frac{\eta_{4}\left(k_{p}-2k_{r1}\right)}{2k_{f1}C+k_{r1}}, & & \\ \gamma & =\frac{2k_{r2}+k_{p}}{\eta_{5}\eta_{7}k_{f2}}\;\delta =\eta_{4}+\beta ,\;\theta =R_{f}N. & \end{array}$$

In all that follows, equation (15) will be used to estimate the concentration of activated VEGF-VEGFR2 dimers in the domain. Adding equations (12) and (14) and substituting in equation (11) gives the following equation for the uptake of VEGF by ECs:

(17) $$\frac{dC}{dt}=-\eta_{0}k_{p}A.$$

Therefore, in equation (10) the cellular uptake function *f*(*p*)*C* = −η_0_*k_p_AI*(*p*), where *I*(*p*) is an indicator function that has a value of 1 at half-lattice sites where EC boundaries are present and is zero otherwise. Observing that η_0_*k_p_* ≪ α*_C_* (see parameter values in Table 2), we make a final simplifying assumption that due to the constant production and rapid diffusion of extracellular VEGF, cellular uptake will not significantly effect its concentration, that is, *f*(*p*)*C* is neglected. Thus, the equation governing free VEGF dynamics is taken to be

(18) $$\frac{\partial C}{\partial t}=D_{c}\Delta C-\alpha_{c}C.$$

**Table 2:** **List of parameter values for single cell motion**.

Parameter	Value	Units	Reference
*D_p_*	1.44 × 10^−4^	mm^2^/h	See text
*D_c_*	3.60 × 10^−1^	mm^2^/h	Mac Gabhann and Popel (2005)
α*_c_*	0.65	Per hour	Serini et al. (2003)
*k_f_1*	1.69	Per (pg VEGF/mm^3^)/h	Wang et al. (2002)
*k_r_1*	0.02	Per hour	Wang et al. (2002)
*k_f_2*	*k_f_1* × 100	Per (pg VEGF-VEGFR2/mm^3^)/h	See text
*k_r_2*	*k_r_1/*100	Per hour	See text
*k_p_*	0.6667	Per hour	Wang et al. (2002)
*R_f_*	0.02	pg receptors per cell face	Stewart et al. (2003), Mac Gabhann and Popel (2004)
*h*	0.02	mm	Vadapalli et al. (2000), Levine et al. (2002)
*k*	0.07	Hours	See equation (5)
χ_0_	0.05	mm^2^ per hour per (pg/mm^3^)^−1^	See text
*K*	2.00	pg/mm^3^	See text

#### 2.3.1 Summary of model equations

The principle variables in our model are: *p*(*n*, *m*, *t*), the probability that a cell occupies lattice site (*n*, *m*) at time *t*; *C*(*x*, *y*, *t*), the concentration of free VEGF at position (*x*, *y*) and at time *t* in pg per lattice site volume, where each lattice site has a height of 1 mm and a base equal to the surface area of a cell; *R*(*i*, *j*, *t*), the concentration of free VEGFR2 at half-lattice sites (*i*, *j*) and at time *t* in pg per lattice site volume; *M*(*i*, *j*, *t*), the concentration of VEGF-VEGFR2 monomers at half-lattice sites (*i*, *j*) and at time *t* in pg per lattice site volume; and *A*(*i*, *j*, *t*), the concentration of free activated VEGFR2-VEGF-VEGFR2 dimers at half-lattice sites (*i*, *j*) and at time *t* in pg per lattice site volume. We remark that *R*, *M*, and *A* can only take positive values at neighboring half-lattice sites where a cell is present, and are 0 otherwise. Equations (1), (7), and (9) that describe the biased random walk of a cell under the influence of activated VEGF-receptors have already been discussed. The following conditions are imposed on the transition probabilities for cell motion as described by equation (1) to ensure that no cell exits the domain:

(19) $$T_{1,m}^{H-}\left(\cdot \right)=T_{N_{s}+1,m}^{H+}\left(\cdot \right)=T_{n,1}^{V-}\left(\cdot \right)=T_{n,N_{s}+1}^{V+}\left(\cdot \right)=0.$$

Here, *N_s_* = 1*/h*, *h* being the lattice size so that 1 ≤ *n*, *m* ≤ *N_s_* + 1.

### 2.4 Parameter estimation

A list of parameter values and sources is given in Table 2. The random motility coefficient of endothelial cells has been estimated to lie within the range 7.2 × 10^−4^–7.2 × 10^−3^ mm^2^/h (Anderson and Chaplain, 1998b). Consequently, intermediate value of 1.44 × 10^−4^ mm^2^/h is assumed. The rates *k_r_1* and *k_f_1* are chosen to ensure that the equilibrium disassociation constant *k_D_* = *k_r_1/k_f_1* has a value of 30.375 pg/mm^3^ (Wang et al., 2002). VEGF binding is known to induce receptor aggregation; therefore, as in Jain et al. (2008) we assume that the rate of formation of a dimerized VEGF-VEGFR2 complex is greater than the rate of formation of a monomer VEGF-VEGFR2 (that is, *k_f_2* ( *k_f_1*). Further, because the dimerized complex *A* is the signaling form of VEGFR2, it is reasonable to assume that *A* is more stable than the monomer complex *M*, that is, *k_r_2* = *k_r_1*. The size *h* of the lattice on which the cell moves is taken to be 20 μm, since typical microvascular endothelial cell volume is about 400 μm (Vadapalli et al., 2000), while its thickness is about 1 μm (Levine et al., 2002). Finally, the parameters χ_0_ and *K* relating to chemotactic sensitivity are chosen to reproduce cell motion and capillary formation profiles that are biologically realistic.

### 2.5 Method of simulation

The time interval over which the movement of the cell is simulated, is divided into subintervals of length *k*, given by the mean waiting time of the cell at any lattice site. The cell moves on a lattice of step-size *h*. Activated VEGFR2 concentrations are calculated at half-lattice sites, neighboring a site where the cell is currently situated. The method of simulation of cell movement is based on that described in Plank et al. (2003). Briefly, at each time step, the movement of the cell is simulated according to the master equation (1), with the probabilities of moving up, down, left, and right calculated according to equation (3). Equation (9) quantifies the dependence of the transition probabilities on the levels of activated VEGFR2 on each cell face as given by equation (15). The full interval [0, 1] is divided into five subintervals, each of length proportional to the probabilities of moving or staying still. A random number *q* lying within this interval is generated, and depending on the sub-interval in which it lies, the cell either executes a motion in the corresponding direction or stays stationary. Thus, the cell moves left if $q\in \left[0,T_{n,m}^{H-}\right),$ moves right if $q\in \left[,T_{n,m}^{H-}+T_{n,m}^{H+}\right),$ and so on.

The results of the single cell motion model are discussed in Section 3.1.

### 2.6 Adaptation of single cell motion model to simulate capillary formation

To simulate capillary formation in response to a VEGF stimulus from a tumor source, we modify the single cell motion model described above as follows. As mentioned earlier, we motivate our model of capillary formation by the experiments of Sholley et al. (1984) wherein inflammatory neovascularization of the rat cornea was induced by cauterization using silver nitrate and levels of EC proliferation and degree of vascular profusion measured periodically. From these experiments, the average rate of sprout extension into the cornea is estimated to be 0.26 mm/day or 0.78 mm in 3 days. In our model, we do not account for vessel maturation; a process that typically occurs after 3 days of vessel formation. Consequently, we simulate vessel growth for lengths ≤0.78 mm. With this constraint, a parent vessel, from which sprout tips will migrate toward the tumor, is assumed at *x* = 0.22 mm. As in the single cell model, a line of tumor cells is assumed at *x* = 1, providing a constant source of VEGF. For ease of computations, the domain size is reduced to 0.5 mm in the *y*-direction, and initially 4 sprouts are assumed to have formed along the parent vessel at *y* = 0.1, 0.2, 0.3, and 0.4 mm.

It is known that specialized ECs situated at the tips of the sprouts, called tip cells, are activated by, and respond to VEGF, by chemotactic migration (Hangai et al., 2002; Gerhardt et al., 2003). We therefore keep track of these leading cells in our simulations. As a tip cells moves, it pulls behind it a developing vessel. Hence, receptors on its tail are made unavailable for binding VEGF at any given time. This eliminates the possibility for the tip cell to back-track. By keeping track of all the lattice sites a tip cell visits, we know the location of the newly formed vessel behind it.

The processes of branch formation and anastomoses formation of loops by capillary sprouts are also included explicitly in our model. At each time step, as the tip cells migrate under the influence of VEGF, probabilities of motion to adjacent lattice sites are calculated. Anastomoses between the tip cell and a sprout may occur if a sprout is present at a site which the tip cell wants to move to. We assume that the probability of tip cell loss as a result of such an event is 1%. Likewise, as in Anderson and Chaplain (1998b), it is assumed that if another tip cell is encountered at a site, only one of these cells continues to grow (with a probability 99%), while the rest of the time, a loop is created with the loss of both cells.

**Figure 5:**
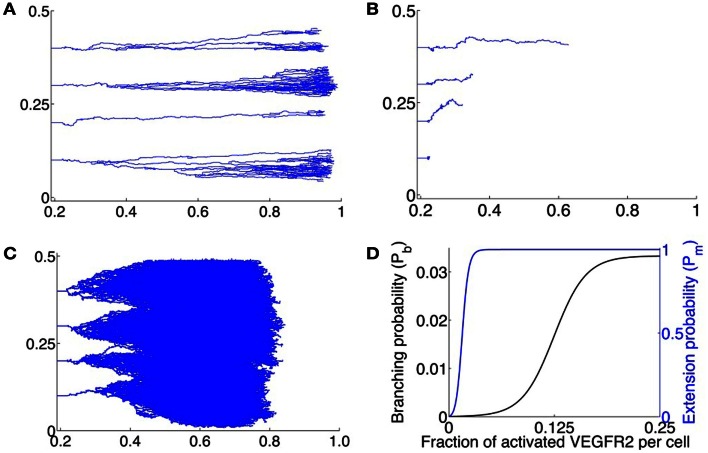
**(A–C)** Typical vascular networks formed by 4 initial sprouts located along *x* = 0.22 at positions *y* = 0.1, 0.2, 0.3, 0.4; *x* being plotted along the abscissa and *y* along the ordinate – migrating across a 2-dimensional domain under the influence of VEGF for various values of *C*_0_, the maximum VEGF concentration per lattice site. **(A)** Optimal VEGF concentration, *C*_0_ = 0.015 pg/lattice volume. The bias of movement is overwhelmingly in the forward direction. Branching and anastomoses are observed to occur as the vasculature penetrates deeper into the stroma. The resulting networks are qualitatively similar to those observed experimentally in Sholley et al. (1984). **(B)** *C*_0_ = 0.005 pg/lattice volume. The amount of VEGF is too low to induce proliferation or polarization of the tip cell, leading to a poorly developed and stunted vasculature that does not reach the VEGF source within the time frame of simulations (3 days). **(C)** *C*_0_ = 0.030 pg/lattice volume. Due to a high VEGF concentration, over-stimulation of endothelial cells occurs, and extensive branching, anastomoses and lateral movement of the tip cell is observed. Due to excessive lateral movement, the vasculature that does not reach the VEGF source within the time frame of simulations (3 days). **(D)** Assumed branching probability *P_b_* of the migrating tip cell (black curve), and assumed extension probability *P_m_* of the capillary (blue curve), expressed as functions of total fraction of activated VEGFR2 per cell.

Sholley et al. (1984) have demonstrated that sprout extension cannot occur in the absence of mitosis. While we do not explicitly model cell division, the dependence of capillary extension on it is accounted for in the processes of capillary elongation and branch formation as follows. The proliferation of cells is known to be regulated by total concentration of activated cell-surface receptors (Gerhardt et al., 2003). Thus, in our model, the tip cell *integrates* the total VEGF bound to it and sprout extension via tip cell motion, and branch formation is only possible if there are enough activated VEGFR2 on its surface. The effect of proliferation on tip cell motion is simulated by introducing a scaling factor of *P_m_*(*A_t_*) that multiplies the movement probabilities of each cell, where *A_t_* is the total concentration of activated VEGFR2 on the cell. *P_m_* is assumed to be a positive, increasing, and saturating function of *A_t_*, with a saturating value of 1. Thus, for small values of *A_t_*, the probability of capillary extension will be ∼ 0 due to an insufficient proliferation stimulus. Here, we take $P_{m}(A_{t})=1/(1+\mu_{m}e^{-A_{t}})$ which is plotted as a function of the fraction of total activated VEGFR2 per tip cell in Figure 5D.

We further assume that the generation of new sprouts occurs only from existing sprout tips. This is in keeping with the fact that there is a region of proliferating cells just behind the tip cell (Sholley et al., 1984), which could give rise to new branches. As in the case for *P_m_*, the branching probability *P_b_* is also taken to be an increasing and saturating function of *A_t_*. This will result in the creation of the brush-border effect. Similar rules for branching have been applied previously by Anderson and Chaplain (1998b). Here, we take *P_b_*(*A_t_*) = 1*/*(μ*_b_1* + *e*^−μ*b*2(*At* − *A*0)^) which is plotted as a function of the fraction of total activated VEGFR2 per tip cell in Figure 5D.

#### 2.6.1 Parameter estimation for capillary formation model

A list of parameter values that are different or new in the capillary formation model is given in Table 3. For consistency with the single cell model, we keep the time step-size *k* unchanged at 0.07 h. Further, the diffusion rate of a tip cell, say *D_t_*, has been estimated to be much smaller than the diffusion rate *D_p_* of an individual EC (Anderson and Chaplain, 1998b; Levine et al., 2001). Therefore, the lattice size *h* for the capillary model needs to be altered accordingly. Equation (5) is used to estimate $h=\sqrt{4kD_{t}}\approx 0.001\text{mm}\text{.}$s Finally, the parameters μ*_m_*, μ*_b1_*, μ*_b2_*, and *A*_0_ relating to the movement probability and branching probability are chosen to reproduce capillary formation profiles that are biologically realistic.

**Table 3:** **List of parameter values for capillary formation**.

Parameter	Value	Units	Reference
*D_t_*	3.60 × 10^−6^	mm^2^/h	(Levine et al., 2001)
*h*	0.001	mm	See text
*k*	0.07	Hours	See text
μ*_m_*	300	Dimensionless	See text
μ*_b1_*	30	Dimensionless	See text
μ*_b2_*	0.25	per pg VEGFR2-VEGF-VEGFR2/mm^3^	See text
*A*_0_	40	pg VEGFR2-VEGF-VEGFR2/mm^3^	See text

#### 2.6.2 Simulation methodology for capillary formation model

The simulation methodology is similar to that of single cell motion described in section 2.5, with the additional computation of accounting for branching and anastomoses for each tip cell, and at each time step. Briefly, in addition to generating a random number *q*, which is used to determine the direction of tip cell motion, two further random numbers are generated (*q_a_* and *q_b_*) by uniformly sampling the interval [0, 1]. We use *q_a_* to determine whether or not anastomoses occurs, and *q_b_* is used to determine whether a new branch forms, in accordance with the rules described above.

The results of the capillary formation simulation are discussed in Section 3.2.

## 3 Results

### 3.1 Single cell motion

Simulations of the system governing a single endothelial cell migrating up a gradient of VEGF, as described by equations (1), (7), (9), and (15), were run in two dimensions, with unbound VEGF profile described by equation (18). The average time in hours it takes for the cell to travel across the domain is plotted in Figures 2C–F, as a function of *C*_0_, the maximum free VEGF concentration at a lattice site, for various possible choices of the chemotactic sensitivity function χ(·). Standard deviations and average times are computed over 500 runs of the model.

**Figure 3:**
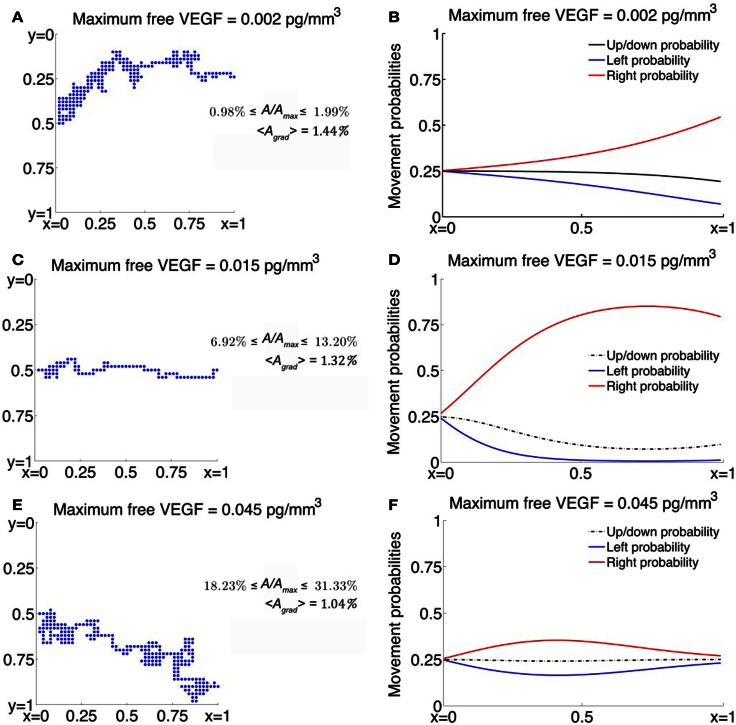
**(A,C,E)** Typical trajectories of a cell migrating across a 2-dimensional domain under the influence of VEGF. Here, *A* represents the concentration of activated VEGFR2 per cell face and *A_max_* represents the maximum value *A* can take so that *A/A_max_* is the fraction of activated VEGFR2 per cell face expressed here as a percentage, and <*A_grad_*> represents the gradient of *A* across a cell length, averaged over the entire domain. **(B,D,F)** Corresponding movement probabilities for various values of maximum VEGF concentration.

Figures 2C,E,F depict the cases when χ(·) is assumed to be a function of activated VEGFR2 on the cell surface. When χ(·) is as defined in equation (8), the model captures the existence of a minimum level of VEGF stimulus required for directed cell motion, as well as desensitization of VEGFR2 at high VEGF concentrations (see Figure 2C). As *C*_0_ increases from 0.002 pg/mm^3^, the average EC migration time is observed to first decrease and then increase, attaining a minimum of 8.23 h at *C*_0_ = 0.015 pg/mm^3^. A typical cellular trajectory is plotted in Figure 3C for this optimal value of *C*_0_, and the corresponding movement probabilities at any lattice site are plotted in Figure 3D. Note that since the VEGF profile is invariant along the *y*-direction, the movement probabilities are also invariant along this axis – they only vary as *x* varies. The probabilities show a large bias toward stepping to the right, while steps to the left are very unlikely to occur. This is because: (i) the chemokine gradient across the cell length has an average value of 1.32%, over the entire domain, which lies within the reported value of 1–2% at which eukaryotic cells become polarized; and (ii) the fraction of activated receptors on any cell face is sufficiently large, with an average value of 9%, over the entire domain.

As *C*_0_ is decreased below 0.015, the average migration time is predicted to increase exponentially. For instance, when *C*_0_ = 0.002 pg/mm^3^, the average migration time is predicted to be 37.46 h, and the cell exhibits a high degree of randomness in its motion, as evident from a typical cellular trajectory shown in Figure 3A. The corresponding movement probabilities at any lattice site plotted in Figure 3B show that a definite bias is apparent for motion to the right only close to *x* = 1. This is because the fraction of activated VEGFR2 on any cell face is very low, with a maximum of < 2%, even though the chemokine gradient across the cell length has an average value of 1.46%. Thus, the model is able to account for the fact that if chemokine concentrations are too low, cell-surface receptors do not achieve a sufficient degree of activation.

As *C*_0_ is increased beyond its optimal value of 0.015–0.08 pg/mm^3^, the model replicates the desensitization effect which has been observed to occur when receptors are over-exposed to chemokines. It now takes the cell an average of 35.77 h to migrate across the domain. From Figure 3E, we observe that typical cell trajectories exhibit a large degree of random motion. Now, activated receptor gradients across the cell have an average value of only 1%. Further, the fraction of activated receptors that vary between 18 and 34% across the domain so that the negative exponential in equation (8) dominates resulting in a very slight bias of movement to the right (see Figure 3F).

For comparison, we also consider the cases where χ(*A*) = χ_0/_(*K* + *A*)^2^ or the receptor-kinetic law and when χ(*A*) = constant = χ_0_. As can be seen from Figure 2E, while the receptor-kinetic law captures the desensitization of VEGFR2 at high concentrations of VEGF, the cell still displays a high degree of directed motion for very low values of *C*_0_. For instance, when *C*_0_ = 0.002 pg/mm^3^ the average migration time is as low as 16.08 h as compared to 37.46 h in the earlier case. In contrast, the existence of a minimum activation threshold for VEGF is predicted by assuming χ(*A*) = χ_0_, as evident from Figure 2F. However, this model is unable to capture receptor desensitization at high values of *C*_0_, and in fact, the average migration time is predicted to decrease monotonically with *C*_0_.

Finally, for illustration purposes, we also consider the case when χ(·) has the same qualitative properties as in equation (8), but the cell now responds to free VEGF rather than activated VEGFR2, that is, χ(*C*) = χ_0Ce_^−*C*/*K*^ (see Figure 2D). While the graph is qualitatively similar to Figure 2C, the fastest migration of the EC across the domain is occurs at *C*_0_ = 7.5 pg/mm^3^. This is biologically implausible since for such high receptor activation levels, the fraction of activated VEGFR2 on any cell face > 0.97 throughout the domain, and the cell should be completely desensitized to the chemical gradient around it.

#### 3.1.1 Effect of receptor expression level on cell migration

**Figure 4:**
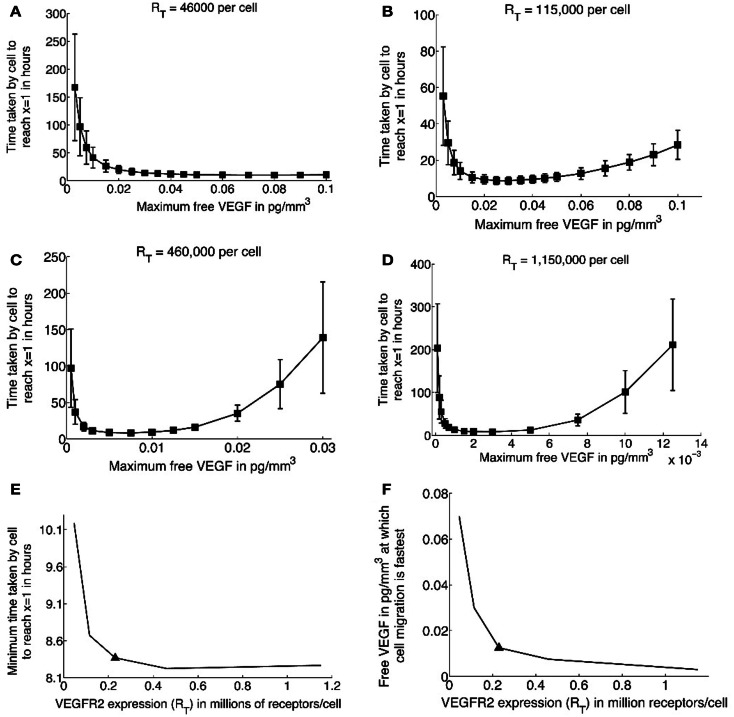
**(A–D)** Average migration times (in hours) for a single cell to travel across a 1 mm × 1 mm domain as a function of increasing the maximum free VEGF concentration at a lattice site, for different values of *R_T_*, the *total* number of VEGFR2 per cell. Baseline simulations correspond to *R_T_* = 230,000/cell, and are shown in Figure 2C. **(E)** Minimum migration time for a single cell to cross the domain as a function of increasing *R_T_*, solid triangle corresponds to baseline simulations. **(F)** The maximum free VEGF concentration at a lattice site (*C*_0_) at which cell migration is fastest, as a function of increasing *R_T_*, solid triangle corresponds to baseline simulations.

An important parameter in our simulations of EC migration is *R_T_*, the expression level of VEGFR2 per cell. This is known to be highly variable across cell lines, and it is even possible to find different values for *R_T_* for the same cell line. We therefore conduct a sensitivity analysis on the migration times of an EC across the domain as *R_T_* is varied, the results of which are graphed in Figure 4. For the baseline simulations discussed earlier, a value of *R_T_* = 230,000 receptors per cell or 0.08 pg/cell (Stewart et al., 2003; Mac Gabhann and Popel, 2004) was used (see Figures 2 and 3). We now simulate the effect on cell migration of increasing *R_T_* from a minimum of 46,000 to a maximum of 1,115,000 receptors per cell for various values of *C*_0_, the maximum free VEGF concentration at a lattice site. For each of the cases when *R_T_* = 115,000 (Figure 4B), *R_T_* = 230,000 (Figure 2C), *R_T_* = 460,000 (Figure 4C), and *R_T_* = 1,150,000 (Figure 4D), the average migration time of the EC is predicted to first decrease and then increase, as *C*_0_ is increases. Thus, for a large range of values of *R_T_*, the model captures the existence of an activation threshold of VEGFR2, and their desensitization when exposed to high VEGF concentrations. However, when *R_T_* is very low (46,000/cell, Figure 4A), receptor desensitization is not predicted. This is possibly due to a high value of the parameter *K*, which is held fixed in all our simulations. As can be seen from equation (8), *K* determines the concentration of activated VEGFR2 per cell face at which the chemotactic sensitivity χ(*A*) is maximum.

Next, as can be seen from a plot of fastest migration times versus receptor expression in Figure 4E, the EC migrates more rapidly across the domain as *R_T_* increases. The fastest migration time is predicted to be 8.25 h, for *R_T_* ≥ 460,000/cell. Interestingly, the maximum free VEGF concentration at which EC migration is fastest decreases with increasing *R_T_* (Figure 4F). Thus receptor over-expression is predicted to lower the activation threshold for ECs, possibly because gradients of activated VEGFR2 become more pronounced across the cell.

### 3.2 Capillary formation

Simulations of the system governing capillary network formation under the influence of VEGF, described in section 2.6, were run in two dimensions. Averages and standard deviations of all observed quantities were calculated from 100 runs of the model.

The first case considered is when the sprout tips move across the domain in the least amount of time. This occurs when the maximum concentration of unbound VEGF *C*_0_ = 0.015 pg/mm^3^, as deduced from the single cell simulations. The results from a typical simulation are shown in Figure 5A. We begin with 4 initially formed sprout tips at *x* = 0.22 mm. As the tip cells migrate across the domain, they lay down behind them capillary sprouts. As the vascular network penetrates deeper into the stroma, branching is observed to occur leading to the brush-border effect. The model predicts that it takes on average 1170 ± 27 steps or 3.38 ± 0.08 days for the vasculature to reach the tumor source at *x* = 1 mm. Our model is validated by the experiments in Sholley et al. (1984) where the vascular sprouts traveled the same distance in 3 days. Further validation follows by observing that the vascular networks generated by our model are qualitatively similar to those observed by Sholley et al. (1984).

Next, the effects of low (0.005 pg/mm^3^) and high (0.030 pg/mm^3^) maximum VEGF concentrations on vascular formation are investigated. As remarked earlier, we do not model vessel maturation, which is typically observed after ∼3 days of vessel formation. Model simulations are run for a maximum of 1170 time steps and the average degree of vascular penetration into the stroma, along with the fraction of sprouts that remain viable (that is, have at least one active tip cell) at the end of this time is computed. When *C*_0_ = 0.005 pg/mm^3^, the average lengths of sprouts formed is predicted to be 0.2 ± 0.1 mm, with only 40% of the initial sprouts still viable after 1170 time steps. Sprouts that remain viable after 1170 time steps extend a greater distance (0.4 ± 0.03 mm) into the stroma. However, these display virtually no branching, with the average number of branches per sprout only 1.1 ± 0.4. Figure 5B shows the results of a typical simulation. As can be seen, there has been no branching and all but the first sprout have anastomosed with themselves to form closed loops. This is due to an insufficient bias to move forwards, coupled with a low value of the scaling factor *P_m_* (see section 2.6).

Finally, when *C*_0_ = 0.030 pg/mm^3^, the average lengths of sprouts formed after 1170 time steps is predicted to be 0.6 ± 0.1 mm. As can be seen from a typical simulation shown in Figure 5C, extensive branching and anastomoses are observed. Given the density of vessel branches, it is reasonable to expect that several of these may fuse into one another resulting in thicker and more dilated vessels, which is a morphology consistent with vascular hyperplasia, as seen in Lee et al. (2005). The higher VEGF concentration implies that the vessels have a weaker bias for forward motion, and lateral movement of vessels as well as movement against the gradient of VEGF are observed to occur. These phenomenon have been observed *in vivo*, and have been numerically simulated previously (Anderson and Chaplain, 1998b; Plank and Sleeman, 2004; Sun et al., 2005; Zheng et al., 2013).

## 4 Conclusion

We have developed a hybrid model of cellular chemotaxis and capillary formation under the influence of VEGF. The migrating cell, whether by itself or as the tip cell “pulling” behind it a developing sprout, was treated as an agent. Its movement was simulated stochastically with movement probabilities based on the theory of biased random walks. On the other hand, due to its fast diffusion coefficient, VEGF dynamics were governed by a continuum reaction-diffusion equation. Using this approach, we first simulated the motion of a single cell on a two-dimensional grid, following the gradient of VEGF laid down by a constant source. Next, our model was adapted to simulate the formation of new vessels from pre-formed sprouts along a parent vessel, also under the influence of a constant source of VEGF, such as a tumor. Events such as branching and anastomoses, which are observed to occur *in vivo*, were incorporated explicitly in the model. The rate of vessel formation closely matched that observed experimentally (Sholley et al., 1984) under an optimal VEGF concentration. Additionally, as the forming vessels neared the VEGF source, a brush-border effect due to increased branching was predicted, thus proving both quantitative and qualitative validation of our approach. Using this framework, we also tested the effects of excessive as well as low levels of VEGF signaling on vascular development. Insufficient chemotactic and mitotic cues from VEGF resulted in stunted and solitary vessels, while an over-stimulation induced a high degree of branching and lateral movement.

An important difference that sets our model apart from similar hybrid models of chemotaxis is the inclusion of a molecular level detail of interaction between VEGF and its cell-surface receptor VEGFR2, the activation of which polarizes the cell and induces directed motion. This has been observed experimentally as well – endothelial cells respond to gradients of chemokines across their lengths, rather than to free chemokine concentrations. These gradients have been shown to be between 1 and 2%, which was seen in the numerical simulations as well, thus validating our model. Crucially, a chemotaxis sensitivity function was proposed that incorporated biological detail hitherto ignored by commonly used sensitivity functions currently. The model could thus capture realistic dynamics, such as the requirement of a minimum activation level of cell-surface receptors and receptor desensitization in high concentrations of VEGF.

Angiogenesis, both physiological and pathological, is a highly complex process, and understanding its mechanisms can lead to significant breakthroughs in the treatment of diseases such as cancer that depend on it. To this end, it is vital that modeling efforts keep up with current advances in experimentation. Our model provides such a framework, in which it is easy to build in biochemical and biomechanical forces guiding vessel formation. In fact, a number of highly detailed and complex hybrid models of vascular tumor growth have recently been proposed (Frieboes et al., 2007; Owen et al., 2009; Perfahl et al., 2011) and a significant strength of our model is that it can easily be incorporated into these. The inclusion of greater biological detail would only increase confidence in the predictive power of such models.

In addition, a number of refinements of the model proposed here are under active consideration. For instance, EC response to cell-surface bound VEGF has already been explicitly included. However, for the ease of computation, certain simplifying assumptions were made. Most notably, activated VEGFR2 were assumed to be in quasi steady state. Further, only the tip cell was tracked, while VEGF uptake by stalk cells was ignored. Cell death was also omitted, while the processes of cell proliferation, branching, and anastomoses were included phenomenologically. We plan to extend this model by relaxing some of these assumptions. Lattice-based models of angiogenesis face the criticism that the capillary networks generated by them are artificial to a certain extent, as they are forced to follow the lattice used to discretize the model. A first step would therefore be to develop a lattice-free version of our model of capillary formation, in which the ECs move without geometric constraints. Such models have been applied to capillary formation previously (Plank and Sleeman, 2004; Frieboes et al., 2007).

Other model refinements include incorporation of the relation between extra cellular matrix or ECM and vascular morphology. ECs require the ECM to gain traction in order to move. To facilitate their migration, ECs also secrete proteolytic enzymes such as matrix metalloproteinases (MMPs), that degrade collagen and elastin and clear a path for the ECs to follow. As ECs interact with the matrix, they also cause the release of matrix bound angiogenic factors such as VEGF, which are then available to induce further pro-angiogenic activity (Mantzaris et al., 2004). Further, pericytes, macrophages, and angiopoietins are also important determinants of developing vascular morphology and maturation (Levine et al., 2000; Plank and Sleeman, 2003), and need to be considered explicitly. The framework presented here is highly flexible, and would allow for the inclusion of the above processes, grounding it further in biology, and enhancing its usefulness as a tool to understanding the process of angiogenesis.

## Conflict of Interest Statement

The authors declare that the research was conducted in the absence of any commercial or financial relationships that could be construed as a potential conflict of interest.
